# A New Intraoral Appliance for Trismus in Oral Submucous Fibrosis

**DOI:** 10.1155/2018/1039391

**Published:** 2018-09-09

**Authors:** Nallan C. S. K. Chaitanya, C. M. S. Krishna Prasad, Reshma Priyanka Danam, Madireddy Nithika, Chintada Suvarna, Jampala Nancypriyanka, Rajkumar Badam

**Affiliations:** ^1^Department of Oral Medicine and Radiology, Panineeya Institute of Dental Sciences, Hyderabad, India; ^2^Department of Orthodontics and Dentofacial Orthopedics, Panineeya Institute of Dental Sciences, Hyderabad, India

## Abstract

Trismus is the most common sequelae of various pathological processes leading to compromised nutritional state in addition to physical and psychological disabilities. Therapeutic interventions are available to relieve trismus, which range from oral usage of pharmacological agents to intralesional steroid therapy. Intraoral appliance therapy can be employed as an alternative or adjuvant treatment for radiotherapy-induced fibrosis and autoimmune disorders such as scleroderma, psychogenic trismus, and oral submucous fibrosis, decreasing the adverse effects associated with other pharmacological interventions. A novel intraoral appliance—“Nallan C-H”—has been developed and tried for trismus producing better results. A report on three such cases having trismus due to a premalignancy has been presented. It is hypothesized that the same appliance can be used for treating inoperable trismus in palliative care setting additionally or as an adjuvant to pharmacological approach.

## 1. Introduction

Trismus refers to the condition where an individual is unable to open the mouth, occurs due to various causes ranging from simple, nonprogressive to potentially life-threatening [1]. It is often considered as a common complication of dental treatment. It has further implications on both mastication and speech [2]. Studies also had shown that trismus is associated with a significant impact on health-related quality of life (HRQOL) [3]. Normal mouth opening in individuals ranges from 40 to 60 mm; in patients with trismus, its restriction varies from a few millimetres (mm) to even several centimetres (cm). For its successful treatment, recognition of its cause followed by initiation of effective management is vital or it may lead to permanent functional impairment [1]. Complications due to radiotherapy include osteoradionecrosis which results in pain, trismus, suppuration, and wound [2]. Most commonly encountered noninfectious causes of trismus are oral submucous fibrosis and radiation-induced fibrosis.

Oral submucous fibrosis (OSMF) refers to chronic, premalignant condition of the oral mucosa. It is prevalent in India affecting 0.2% to 0.5% of the general population with gender variation of 0.2–2.3% in males and 1.2–4.57% in females. Age distribution of patients with OSMF is wide, ranging between 20 and 40 years. The initial presentation of OSMF is inflammation, followed by hypovascularity and fibrosis. Moderate stage of OSMF (group II and group III by Khanna et al.) is characterised by irreversible fibrosis which is progressive, invariably leading to trismus with variable mouth opening. Trismus in oral submucous fibrosis is due to fibrosis of the lamina propria resulting in loss of elasticity and stiffness. It is different in other conditions such as space infections, muscle spasm due to tetanus, maxillomandibular factures in which collagen fibres are unaffected, and no loss of elasticity. OSMF-related trismus has further bearing on oral hygiene, speech, mastication, and possibly swallowing. Moreover, the risk for its malignant transformation is found to be varied from 7% to 30% [4].

Along with other treatment modalities such as intralesional corticosteroids with and without combination of placentrex, surgical release of the fibrous bands is another treatment modality for this condition. Relapse is the common complication during and after treatment, and the compliance with treatment is a major detrimental factor for successful outcome. Alternative options in treating are pentoxifylline 400 mg thrice daily medication, vitamin A supplements, heat short-wave diathermy, antioxidants, immunised milk, turmeric, and aloe vera oral applications. All of these modalities have limitations and relapse [5].

An intraoral appliance which would not be cumbersome and bulkier in oral cavity and also comfortable for the patient to wear would be beneficial. The appliance should not demonstrate functional changes in the teeth as well as in the occlusion. It should cause desirable mouth opening with minimal side effects. This case series focuses on the development and fabrication of a novel intraoral appliance called “Nallan C-H appliance” for the improvement of mouth opening in patients with trismus. It is noninvasive, economical, and had fair compliance with the patients.

## 2. Case Reports

### 2.1. Case 1

**Figure 1:**
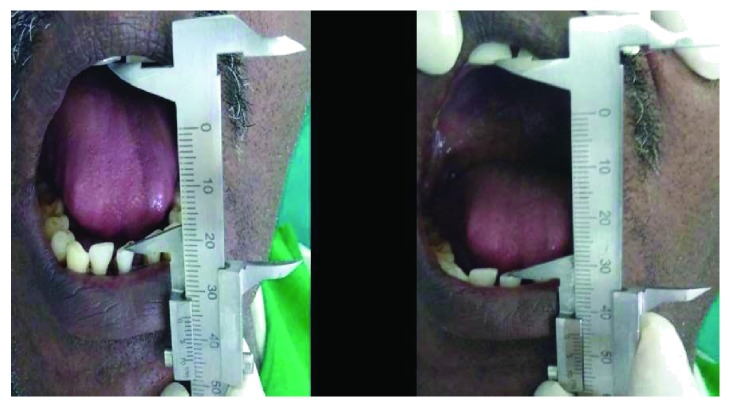
Pre and post appliance therapy.

A male patient, aged 39 years, presented to a private clinic with a chief complaint of difficulty in mouth opening since one and half years. The patient had a habit of chewing *gutka* for the past eight years. It was observed that there is noticeable decline in mouth opening of 17 mm (intercanine distance) and tongue protrusion of 10 mm. On intraoral examination, generalized blanching of the oral mucosa with grayish black pigmentation was seen. And also, multiple vertical palpable fibrous bands with loss of elasticity and leathery in texture were noticed. OSMF was diagnosed, and the patient was treated with conventional intralesional steroid injections. Since the patient has been under similar treatment for over a period of time with no recognisable change or relief, he requested for an alternative therapy. Intraoral appliance therapy was considered, and prior consent was obtained from the patient. The patient was duly provided with necessary precautions regarding the usage of appliance and weekly follow-up without discontinuing the treatment. The treatment was carried out for a total period of 8 weeks and a follow-up of two months after completion of the therapy (Figure 1).

### 2.2. Case 2

A female patient, aged 56 years, presented to the private clinic with chief complaint of difficulty in mouth opening since one month. During her first visit, i.e., approximately a year back, she reported about the treatment that she received for trismus (due to OSMF) using intralesional injections. At that time, the patient had marginal relief from the symptoms. Again, she started developing trismus since one month and also had burning sensation in the oral cavity. Patient had restricted mouth opening of 30 mm (canine-canine distance) and tongue protrusion of 12 mm with all signs of OSMF (group 2 by Khanna et al.) in the oral cavity. As she was not able to tolerate any more pain from intralesional steroid injections, she was advised intraoral appliance therapy for 8 weeks. She was also instructed for weekly follow-ups with precautions during appliance position in the oral cavity.

### 2.3. Case 3

A male patient, aged 40 years, with a history of chewing betel quid for the past 15 years, presented to the private clinic with reduction in mouth opening since one year. Patient had a restricted mouth opening of 35 mm (canine-canine distance) and tongue protrusion of 12 mm with all signs of OSMF (group 2 by Khanna et al.) in the oral cavity. The patient was then started with intralesional corticosteroids, which showed improved mouth opening till 42 mm (canine-canine distance), and then this treatment modality was discontinued due to pain arising from repeated punctures. The patient then requested for alternative therapy. He was advised appliance therapy and was instructed for weekly follow-ups for 8 weeks with precautions in positioning and usage of the appliance in the oral cavity.

## 3. Clinical Procedure and Fabrication of the Appliance

For all patients, maximum mouth opening was recorded using appropriate measuring device at baseline prior to the initiation of the appliance therapy. Necessary precautions were taken during the fabrication of the appliance such as the following:

1. The appliance should not impinge gingival margins
2. It should be easy to manipulate by the patient
3. Should be comfortable to use and also rigid enough to resist masticatory forces

**Figure 2:**
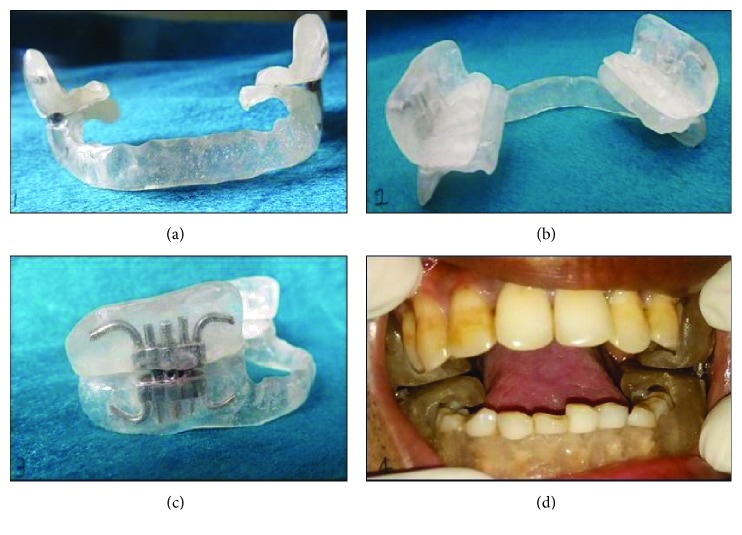
(a) Frontal view of the appliance with labial extension in the lower arch. (b) Posterior view of the appliance with occlusal plates and buccal extensions in both upper and lower arches. (c) Lateral view of the appliance with hyrax screws placed within the acrylic plates. (d) Intraoral placement with activated appliance and separation in progress.

Alginate impression was taken for both upper and lower arches with stock metal trays. Impression was poured using dental stone. The obtained casts were then articulated with an apex articulator. Fabrication of appliance was done by using self-cure acrylic resin and sprinkle on technique covering the sulcus area in the anterior region, which broadens posteriorly to cover the buccal area and occlusal surface of the lower arch. On the upper arch, only the molar area covered the teeth both occlusally and buccally. Mounting of cast in the occlusion was done in a hinge articulator so that occlusal relation was maintained. The wax was then adapted on the buccal surface of the lower arch to keep the distance of 2 mm from the gingiva, so that it did not impinge the soft tissues. Hyrax screws of 12 mm gauge were adapted bilaterally on the buccal aspects of the molars on the wax. Precaution was taken to avoid blocking the activation hole of the screws. Once the acrylic had begun to set, the appliance was removed cleaned, trimmed, and polished. A lower labial extension was given for the appliance in order to prevent accidental breakage and subsequent aspiration of the appliance (Figure 2).

The appliance was then tried in the patient's mouth and adjusted according to his/her convenience. Care was taken to avoid excess pressure. The patients were educated regarding proper insertion, removal, and maintenance of the appliance and oral care. Moreover, they were encouraged to wear the appliance 12 hours overnight for 8 weeks and followed up every week to check any improvement. Patients were also encouraged to perform isometric mouth exercises daily according to their comfort. For every visit, the mouth opening was measured and the screw was released 1 mm on each side to improve mouth opening. A follow-up of 2 months was performed on each patient (Table 1).

## 4. Treatment Evaluation and Follow-Up

It was observed that there was significant increase in mouth opening in all three patients ranging from 2 to 8 mm. None of the patients reported difficulty in the placement of appliance in the oral cavity during the treatment phase. No significant decrease in mouth opening was observed during post appliance follow-up of 2 months. However, there was a decrease of 2 mm in the third patient and 0.5 mm in the first patient.

## 5. Discussion

Trismus is defined as a prolonged tonic spasm of the muscles, which results in restricted mouth opening. OSMF is a potentially malignant, chronic, progressive disorder seen mostly in people from Asia and is found to affect most of the parts of the oral cavity that includes the lips, tongue, palate, pharynx, and even the upper third of the oesophagus. In later stages, further stiffening occurs due to myofibrosis of the subepithelial and submucosal tissues, thereby resulting in limitations in the mouth opening and tongue protrusion causing difficulty in eating, swallowing, and also phonation-related issues [6]. Various treatment modalities such as physical oral therapy, intralesional corticosteroids, ultrasound therapy, and surgical modalities were tried till date [4].

In the present case series, a newer treatment procedure is tried on patients suffering from OSMF. An appliance that can be easily fabricated was designed and used in patients with trismus due to any noninfectious pathology. For patients who are not comfortable or given consent for treatment with intralesional steroids, this appliance therapy could be an alternative treatment modality. Moreover, cost and the adverse effects are involved in this treatment when compared with steroid therapy. Yadav et al. believed that protecting surgically reconstructed defects using flaps is vital, and the authors fabricated an appliance in order to avoid trauma to the flap in the postoperative period [6]. It is further believed that physiotherapeutic effect is a probable mechanism behind appliance therapy, which causes remodelling of the tissues for improving mouth opening [7].

From design, the appliance works by causing mechanical force which then induces the stretching of the elevator and depressor muscles. Based on the design, these appliances are classified into externally and internally activated types. Externally activated appliances exert force by stretching the elevator muscles and depressing the mandible whereas internally activated appliances employ the force on the depressor muscles to stretch the elevator muscles. They impart forces which are continuous or intermittent, elastic or nonelastic, and light or heavy. The force generated by the elevator muscles is greater than that by the depressor muscles. The amount of force delivered depends on the strength, frequency, duration of stretching, and motivation of the patient [8].

The activation cycle is unique to the appliance. The key which is provided for opening up the hyrax screws during rapid palatal expansion is used for activation purposes in relieving the trismus. Each full turn is equal to approximately 0.2 mm and total number of 4 full turns is given which account to 0.8 mm per week. The patient is followed up every week for 8 weeks. The approximate mouth opening hypothesized ranges from 5 mm to 1.5 cm.

In the present case series, the appliance emits intermittent and bilateral forces, which help to depress the mandible and make the maxillary and mandibular teeth apart thereby relieving the trismus. Physical therapy improves the range of motion of temporomandibular joint, reduces pain, prevents hypomobility, avoids fibrosis formation, strengthens the musculature, and improves flexibility, tissue elasticity, and blood circulation.

The appliance can be fabricated in patients who are completely edentulous and also in those partially edentulous patients. As the appliance is passive over the teeth and does not cause functional tooth movements, it can be comfortably worn in patients with missing dentition. As the occlusion is undisturbed, there is elimination of alteration in the occlusion sequence. Caution has to be maintained when there are periodontal compromised teeth. Excessive vertical forces may further breakdown the periodontium. Periodontal assessment has to be carried out before the appliance fabrication. Teeth with excessive mobility and poor prognosis should be managed prior to appliance insertion. Mild-to-moderate periodontitis is not a contraindication for appliance fabrication.

Patil et al. from their study concluded that the use of mouth exercising device appears to be effective for the separation of collagen fibers and increased the subcutaneous matrix area leading to improved blood circulation [9]. Oswal et al. fabricated an oral screen prosthesis to stabilize the secured flaps and to prevent it from being bitten into occlusion, and the same can also be used as an oral stent to prevent relapse [10]. Similarly, Li et al. fabricated a EZBite open mouth device and conducted a 12-week structured open mouth training program and stated a marked improvement in mouth opening [11].

Till date, there are two jaw-exercising devices used in palliative care. “TheraBite” is a mechanical device with lever system which assists mouth opening by squeezing the handle of the device and is able to control the extent of the stretch to the tissues. Another appliance is “Dynasplint trismus system” which is used with a low-torque and prolonged duration stretch designed to lengthen connective tissue [12, 13]. Both of these appliances are used effectively to relieve trismus due to various causes. TheraBite is a lever system which is patient dependent, and the maximum opening claimed is almost 41 mm. Dynasplint trismus system is bulkier compared to the presently described appliance. The present appliance is not visible outside the oral cavity unlike the two above-described systems. The mandibular range of motion may not be achieved with the present model, and modifications may be required to assess the same in further fabrication.

## 6. Biocompatibility of the Appliance

The appliance is made up of acrylic resin material which is routinely used in the fabrication of partial or complete removable dentures and hyrax screw which may be used for palatal expansion in orthodontic treatment. The components have been proven to be biocompatible in the patients. The labial extension of the appliance is a safety measure which enables the appliance to stay fit in the oral cavity without breakage and accidental slip into the oral mucosa and esophagus.

## 7. Limitations and Adverse Effects

The adverse effects with present appliance were as follows:

1. Difficulty to insert intraorally during the initial phases of treatment
2. Excessive salivation
3. Reduced strength of appliance after weeks of usage may be due to fabrication errors

## 8. Conclusion and Future Recommendations

The present appliance can also be successfully intervened in trismus arising from any of the abovementioned causes without much adverse effects. Long-term studies are required to evaluate the effectiveness of the appliance over a large group of population and its compliance among the patients. Follow-up at regular intervals may be required for effective management of the patients with trismus and relapse.

## Figures and Tables

**(a):** 

Phase 1	(At diagnosis)
Cases	Mouth opening (mm)
Case 1	17
Case 2	30
Case 3	35

**(b):** 

Phase 2	(After complete treatment)
Cases	Mouth opening (mm)
Case 1	22.5
Case 2	32
Case 3	45

**(c):** 

Phase 3	(Follow-up after 2 months)
Cases	Mouth opening (mm)
Case 1	22
Case 2	32
Case 3	42
